# Pure Sertoli cell tumor of the right ovary managed with fertility-sparing surgery: a case report

**DOI:** 10.1093/omcr/omag131

**Published:** 2026-07-12

**Authors:** Yohannis Derbew Molla, Suleiman Ayalew Belay, Abebaw Muhabaw Zegeye, Samuel Addisu Abera, Endeshaw Asaye Kindie, Getachew Shiferaw Yigezaw

**Affiliations:** Department of Surgery, College of Medicine and Health Sciences, University of Gondar, Maraki Street, Gondar City, Central Gondar Zone, PO Box 196, Gondar, Ethiopia; School of Medicine, College of Medicine and Health Sciences, University of Gondar, Maraki Street, Gondar City, Central Gondar Zone, PO Box 196, Gondar, Ethiopia; Department of Surgery, College of Medicine and Health Sciences, University of Gondar, Maraki Street, Gondar City, Central Gondar Zone, PO Box 196, Gondar, Ethiopia; Department of Pathology, College of Medicine and Health Sciences, University of Gondar, Maraki Street, Gondar City, Central Gondar Zone, PO Box 196, Gondar, Ethiopia; Department of Pathology, College of Medicine and Health Sciences, University of Gondar, Maraki Street, Gondar City, Central Gondar Zone, PO Box 196, Gondar, Ethiopia; Department of Obstetrics and Gynecology, College of Medicine and Health Sciences, University of Gondar, Maraki Street, Gondar City, Central Gondar Zone, PO Box 196, Gondar, Ethiopia

**Keywords:** sex cord stromal tumor, Sertoli cell tumor, ovarian neoplasm

## Abstract

Sertoli cell tumors (SCTs) of the ovary are rare sex cord–stromal neoplasms. We describe a 37-year-old Ethiopian woman (G2P2) with six months of progressive lower abdominal pain but no virilization. Ultrasound revealed a solid 11 cm right adnexal mass. Hormonal profile was normal. An open fertility-sparing right salpingo-oophorectomy was performed. Histopathology confirmed a FIGO Stage IA well-differentiated pure SCT. Postoperative surveillance was conducted via clinical pelvic examinations and abdominal/pelvic ultrasounds every three months. At 12-month follow-up, there was no recurrence. This case highlights that SCTs may be hormonally inactive; histopathologic evaluation remains decisive, and fertility-preserving surgery is a safe option in early-stage lesions.

## Introduction

Ovarian Sertoli cell tumors are extremely uncommon, representing approximately 0.1–0.5% of ovarian neoplasms [1, 2]. They typically affect reproductive-age women. Diagnosis and management are guided by the International Federation of Gynecology and Obstetrics (FIGO) staging system. Imaging modalities, including pelvic ultrasound, computed tomography (CT), and magnetic resonance imaging (MRI), are crucial for preoperative characterization, though SCTs often appear as non-specific solid masses [3]. While many secrete androgens, leading to virilization, others are hormonally silent [4]. We present a well-differentiated, hormonally inactive pure SCT managed with fertility-sparing surgery and discuss diagnostic and management considerations with reference to existing literature.

## Case presentation

A 37-year-old Ethiopian female (G2P2) patient presented with a complaint of lower abdominal dull ache and dragging sensations that had been persisting for six months. Notably, she did not experience any swelling, nausea, vomiting, or changes in bowel movements. Her menstrual history was regular, with no abnormal bleeding, and there were no signs of hyperandrogenism or hyperestrogenism. There was no family history of breast, ovarian, or colonic malignancy. The patient had no significant past surgical history or history of trauma, and she did not have any chronic diseases such as diabetes or hypertension. Furthermore, she had no known drug allergies.

During the examination, all vital signs were found to be within normal ranges, and her Body Mass Index (BMI) was calculated to be 23 kg/m^2^. There was no tenderness in the abdomen, and no palpable mass was detected. However, a bimanual pelvic examination revealed an ill-defined mass in the right adnexa, while other examinations showed no remarkable findings. Baseline hematologic and biochemical tests were normal. Hormonal assays showed total testosterone 28 ng/dl (ref 15–70), DHEA-S 160 μg/dl (35–430), estradiol 82 pg/ml (follicular 20–150), LH 6 IU/l, FSH 5.2 IU/l, prolactin 12 ng/mL all within institutional limits. Pelvic ultrasound demonstrated an 11 × 9.5 × 8.8 cm predominantly solid, lobulated right adnexal mass with internal vascularity (RI 0.48), no ascites, and no suspicious lymphadenopathy. Cross-sectional imaging (CT/MRI) was not performed due to limited local availability of advanced imaging at the time of diagnosis.

Based on the assessment of the right adnexal mass, the patient underwent an open right salpingo-oophorectomy via midline laparotomy. Surgery. Intraoperatively, a completely solid mass was found in the right ovary, characterized by a creamy color and an intact capsule, with no presence of ascites or lymphadenopathy. There were no signs of metastasis or tumor deposits. Consequently, a unilateral right salpingoophorectomy was performed. During the procedure, 100 ml of normal saline was used to perform peritoneal washings, which were sent for cytopathological examination. The cytopathology report subsequently confirmed the absence of malignant cells. Macroscopically, the tumor was confined to the right ovary with a smooth, intact capsule and no surface excrescences. Systematic exploration of the peritoneal surfaces, omentum, and contralateral ovary showed no evidence of metastasis. Based on these findings, the tumor was staged as FIGO Stage IA.

Following the surgery, the patient received diclofenac intramuscularly (IM) twice a day for pain management, along with daily wound care. After a three-day hospital stay with no perioperative complications, the patient was discharged and given an appointment at the referral clinic. Postoperative surveillance was established every three months for the first year, including clinical pelvic examinations and abdominal/pelvic ultrasounds to monitor for recurrence. Hormonal levels remained stable, and the patient remained asymptomatic with no evidence of disease at the 12-month mark.

**Figure 1:**
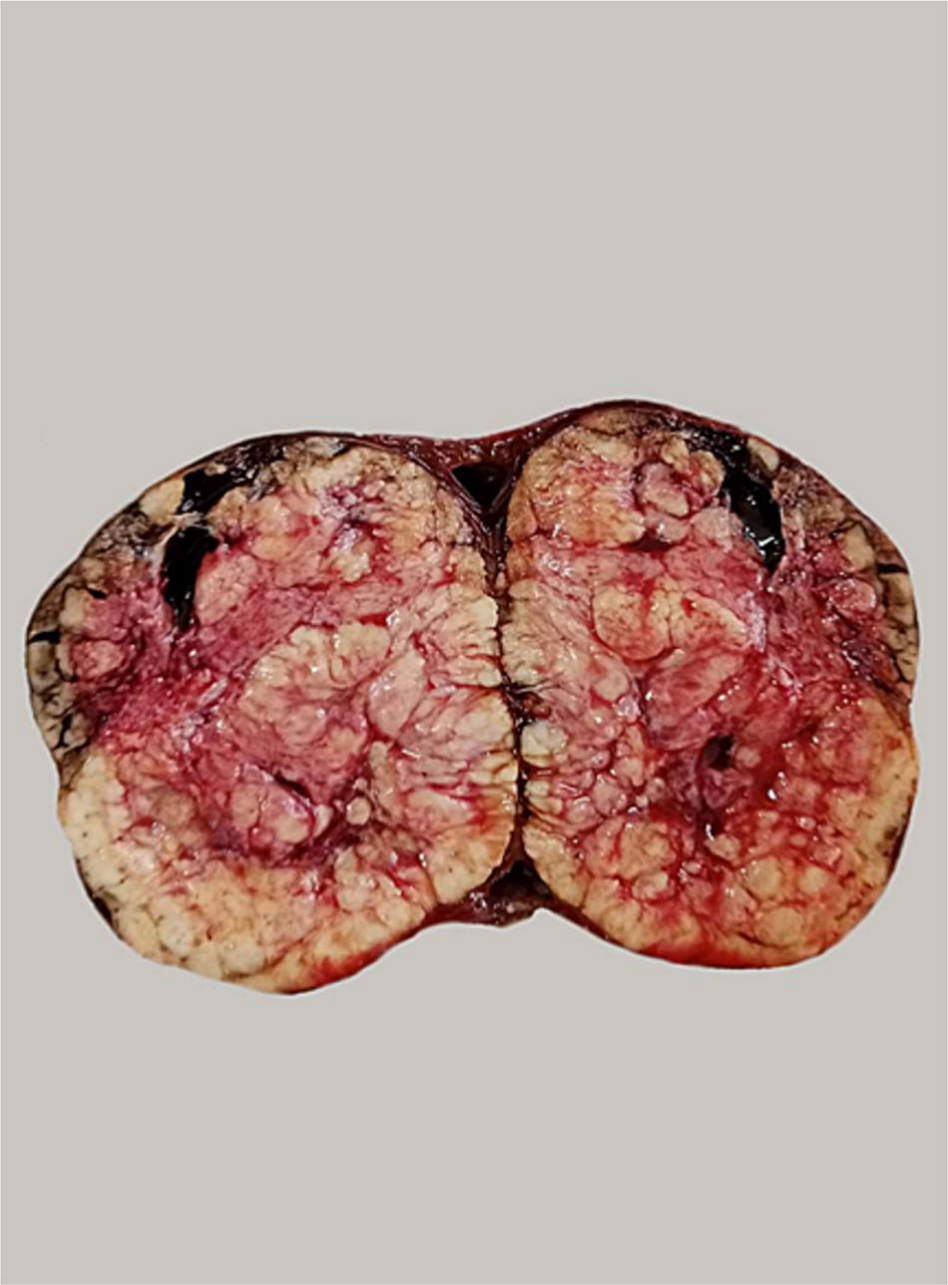
Gross cut section showing a solid, yellowish, lobulated ovarian mass with an intact capsule.

**Figure 2:**
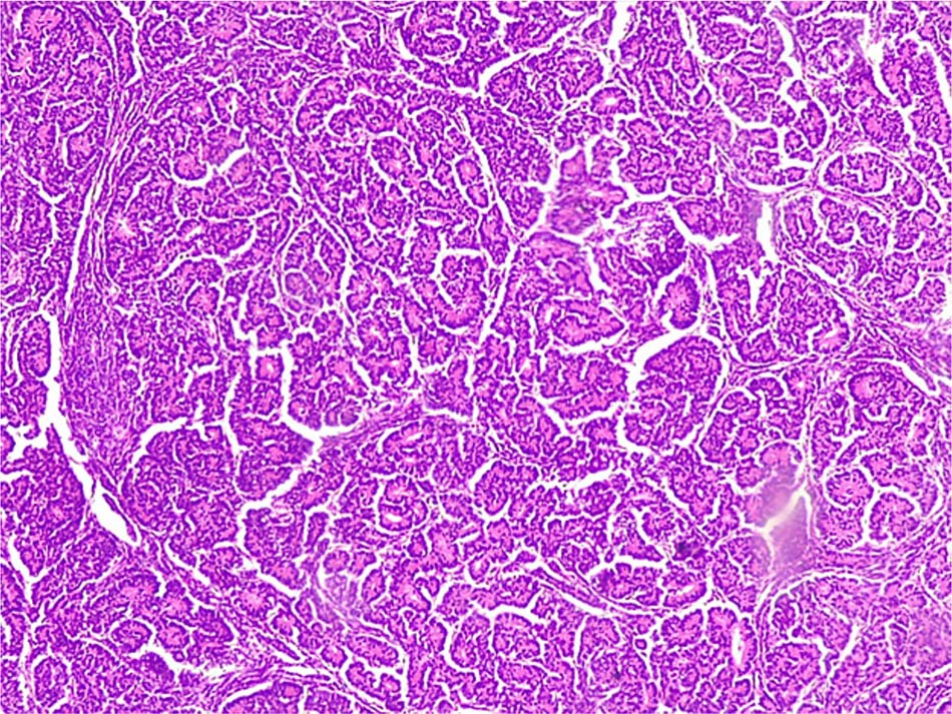
Histopathology (100×) showing a distinctive tubular architecture with structures vaguely lobulated by scant fibrous stroma.

**Figure 3:**
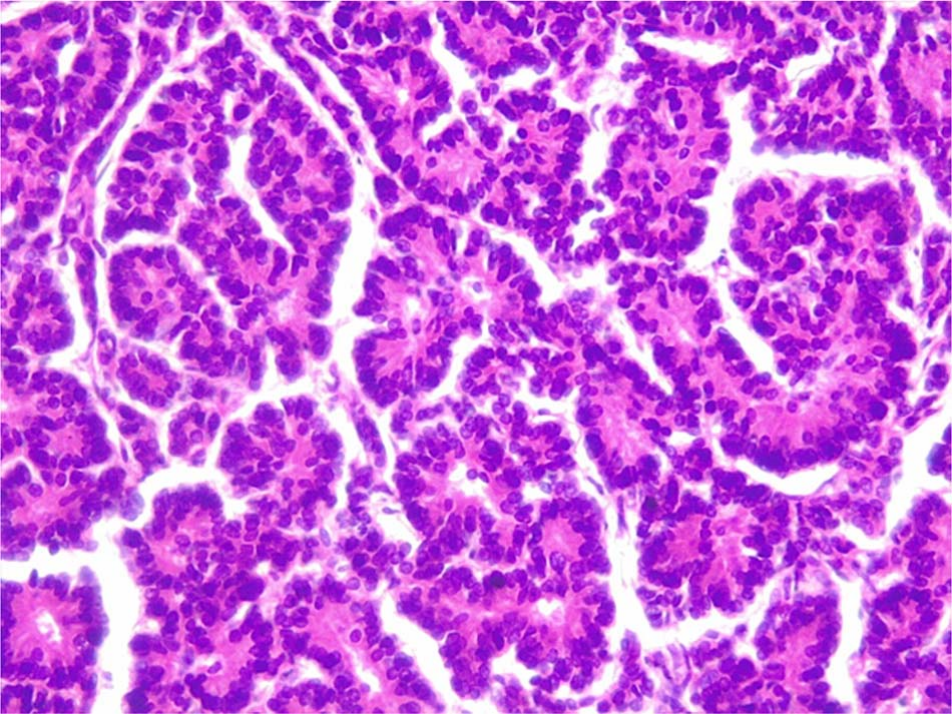
Histopathology (200×) showing anastomosing solid and hollow tubules characteristic of Sertoli cell differentiation.

**Figure 4:**
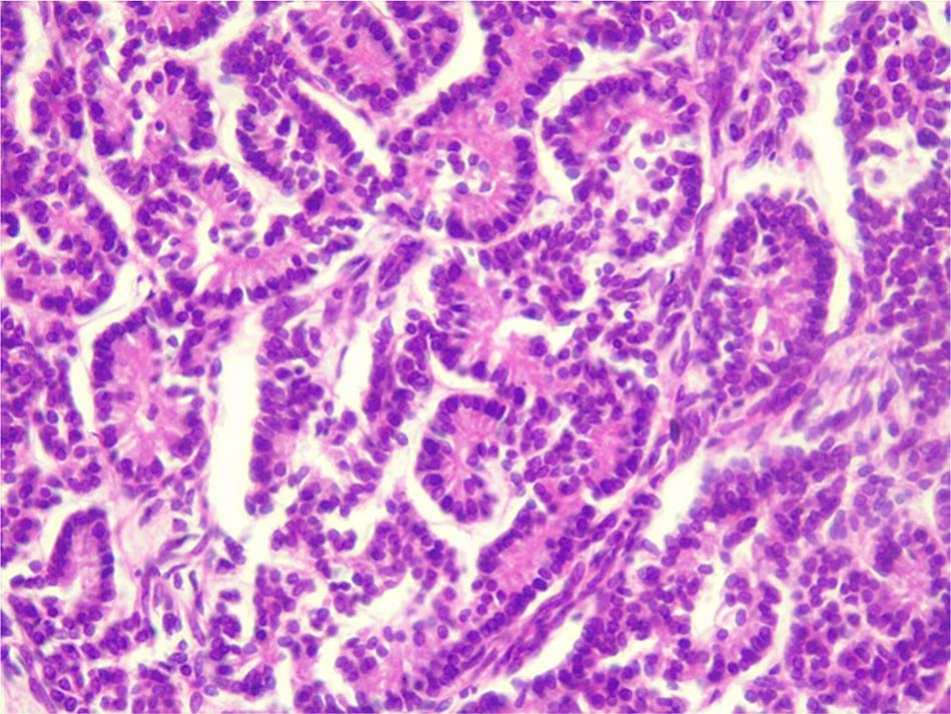
Histopathology (400×) showing high-power detail of uniform columnar cells with basal round-to-oval nuclei and eosinophilic apical cytoplasm; no mitotic figures are identified.

**Figure 5:**
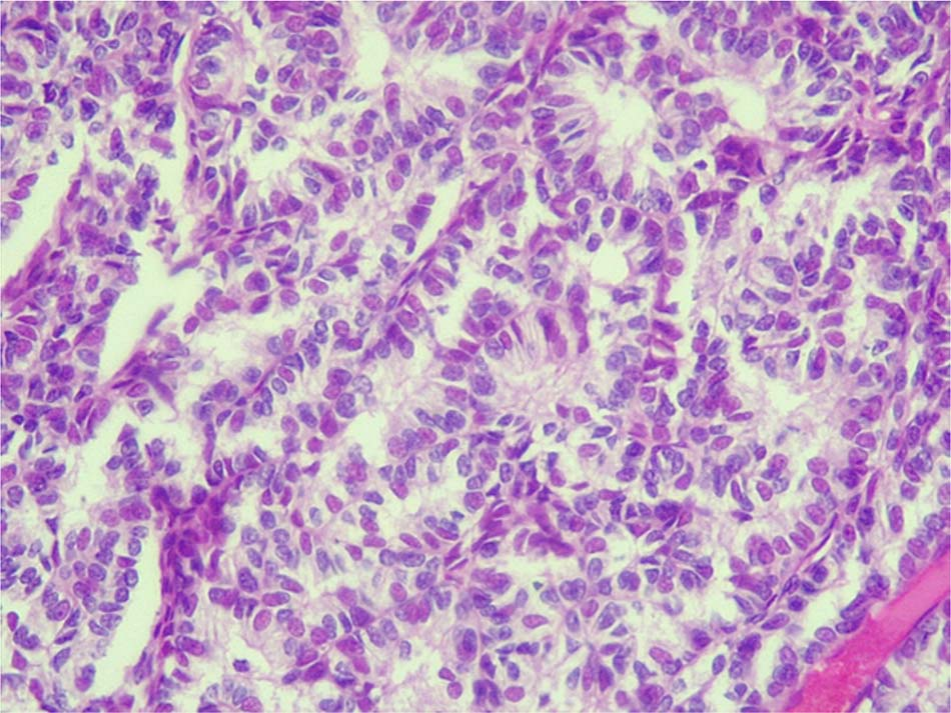
Histopathology (400×) showing a focal area of cytoplasmic clearing, a known morphological variant of SCT.

The gross examination of the excised mass revealed a completely solid ovarian mass with a lobulated appearance on the cut surface, which appeared yellowish in color (Fig. 1). Upon further examination of histopathology sections, it was observed that the mass consisted of both hollow and solid tubular structures composed of columnar cells. These cells exhibited basal nuclei, uniform chromatin, and moderate to abundant eosinophilic apical cytoplasm. The tubes appeared vaguely lobulated due to a small amount of fibrous stroma. Additionally, a focal area of cytoplasmic clearing was observed (Figs 2–5). Despite extensive sampling, no Leydig cells were identified, and there was no evidence of cytologic atypia, mitosis, or necrosis.

## Discussion

Pure ovarian SCTs are rare variants within the sex cord–stromal category, distinct from Sertoli–Leydig tumors (SLCTs) by absence of Leydig elements [1, 5]. They often present in the third to fifth decades, either hormonally active or, as in our case, completely inactive [6, 7]. Normal serum testosterone and estradiol levels support a non-functional phenotype, which can delay diagnosis when symptoms are limited to mass effect.

Imaging findings for SCTs are often nonspecific, typically presenting as solid or mixed solid–cystic masses with internal vascularity [3, 8]. While histopathology remains the diagnostic cornerstone, immunohistochemical (IHC) markers—specifically inhibin, calretinin, and SF-1—are considered the gold standard to confirm sex-cord differentiation [4, 9]. In resource-limited settings where such ancillary studies may be unavailable, a definitive diagnosis relies on meticulous morphologic evaluation. In our case, the diagnosis of a pure SCT was confidently established by the pathognomonic presence of well-defined hollow and solid tubules. This classic architecture, combined with the total absence of Leydig or granulosa cells, provided sufficient evidence for a definitive diagnosis despite the lack of IHC.

The differential diagnosis for a pure Sertoli cell tumor is broad, encompassing Sertoli–Leydig cell tumors (SLCTs), adult granulosa cell tumors (AGCTs), sertoliform endometrioid carcinomas, and metastatic neoplasms. In this case, SLCTs were ruled out by the total absence of Leydig cells and pathognomonic Reinke crystals, a finding further supported by the patient’s normal serum androgen levels [5]. AGCTs were excluded based on the lack of characteristic ‘coffee-bean’ nuclear grooves and Call-Exner bodies. While endometrioid carcinomas can mimic the sertoliform pattern, the absence of typical endometrioid glandular components or squamous metaplasia pointed toward a primary sex-cord tumor [10]. Finally, the unilateral presentation and classic tubular morphology, combined with the lack of a known extra-ovarian primary, made metastatic carcinoma highly improbable.

Surgical excision remains the cornerstone of management for ovarian SCTs. For early-stage, well-differentiated neoplasms—such as this FIGO Stage IA case—unilateral salpingo-oophorectomy is considered both diagnostic and therapeutic, offering curative potential while successfully preserving fertility in reproductive-age patients [11, 12]. The favorable prognosis in our patient is further supported by the histopathologic absence of necrosis, cytologic atypia, and mitotic activity, all of which are strong predictors of a benign clinical course. While long-term survival for well-differentiated Stage I tumors exceeds 95% [7, 12], we emphasize the necessity of regular postoperative surveillance with clinical examinations and pelvic imaging, as late recurrences, though rare, have been documented in the literature [13]. In contrast, advanced or poorly differentiated tumors may necessitate more aggressive intervention, including formal staging laparotomy and adjuvant platinum-based chemotherapy [14].

Recent literature emphasizes the clinical variety of these tumors; for instance, Bužinskienė et al. (2022) reported a case with severe hyperandrogenism and elevated testosterone, whereas our patient remained normo-androgenic [15]. Furthermore, Shrestha et al. (2022) described a case in a 46-year-old woman managed with total hysterectomy; in contrast, our case demonstrates that fertility-sparing surgery is viable for younger patients with Stage IA disease [16].

A major limitation in our setting was the lack of immunohistochemistry (IHC) and advanced cross-sectional imaging (CT/MRI). IHC markers such as inhibin, calretinin, and SF-1 are the gold standard for confirming sex-cord differentiation [4]. In their absence, our diagnostic confidence relied on the pathognomonic morphology: well-defined hollow and solid tubules lined by columnar cells with uniform basal nuclei. While the lack of preoperative CT/MRI may limit the detection of occult lymphadenopathy, thorough intraoperative staging and the well-differentiated nature of the tumor mitigated this risk. We also acknowledge that our 12-month follow-up is relatively short; since late recurrences can occur, continued long-term surveillance is mandatory [13].

Our case demonstrates successful management of a hormonally inactive, localized pure SCT using a fertility-sparing approach. The absence of IHC was a limitation but did not hinder accurate diagnosis due to classic morphology and benign features.

## Conclusion

Pure Sertoli cell tumor of the ovary is a rare neoplasm that can present without endocrine symptoms. In well-differentiated, early-stage cases, unilateral salpingo-oophorectomy ensures disease control while preserving fertility. Careful histopathologic evaluation and long-term follow-up are essential, especially in settings lacking immunohistochemistry.
